# IUC24430-78 Lesion-level meta-diagnostic of cognitive vs fusion MRI-targeted biopsy in PCa: PRECISION-2 insights

**DOI:** 10.1093/oncolo/oyaf248.007

**Published:** 2025-08-29

**Authors:** R Daniswara, M Amin, R Zaidan

**Affiliations:** University of Indonesia, Indonesia; University of Indonesia, Indonesia; University of Indonesia, Indonesia

## Abstract

**Background:**

Prostate cancer (PCa) is among the most common malignancies globally, with increasing cases in both developed and emerging regions. Early, accurate detection is essential for risk assessment, treatment planning, and avoiding over-treatment. MRI-targeted biopsy has become vital, using 2 main techniques: cognitive fusion (COG-TB), where operators visually match MRI with ultrasound, and software-based fusion (FUS-TB), which overlays MRI onto ultrasound via specialized platforms. To compare the diagnostic accuracy, which consists of sensitivity, specificity, and area under the curve of COG-TB and FUS-TB for detecting prostate cancer through a meta-diagnostic test.

**Methods:**

A random-effects meta-analysis was performed using data from PubMed, Scopus, and Cochrane following the PRISMA 2020 guidelines. Statistical analysis was conducted using the R{mada} package with a 95% CI. Diagnostic performance is presented through the area under the curve (AUC), sensitivity, and specificity. Heterogeneity was assessed using the Higgins methods.

**Results:**

COG-TB has the sensitivity level of 66.9%, specificity of 59.9%, and AUC of 0.672. On the other hand, FUS-TB has the sensitivity level of 74.5%, specificity level of 56.3%, and AUC of 0.739. Comparing both results, FUS-TB shows a better result in identifying people with prostate cancer, which is supported by higher sensitivity and AUC score. However, COG-TB has higher accuracy in terms of detecting persons without prostate cancer, which is supported by higher specificity.

**Conclusions:**

The meta-diagnostic FUS-TB is a better tool in terms of detecting people with prostate cancer. COG-TB, with a higher specificity score, poses as a better tool in detecting people without prostate cancer. These results might become a consideration to further develop both of the tools to maximize the potential in terms of prostate cancer diagnosis.

